# Patient-centred goal setting using functional outcome measures in geriatric rehabilitation: is it feasible?

**DOI:** 10.1007/s41999-017-0011-5

**Published:** 2017-12-21

**Authors:** Ewout B. Smit, Hylco Bouwstra, Johannes C. van der Wouden, Lizette M. Wattel, Cees M. P. M. Hertogh

**Affiliations:** Department of General Practice and Elderly Care Medicine, Amsterdam Public Health Research Institute, VU University Medical Center, Van der Boechorststraat 7, Room H465, 1081 BT Amsterdam, The Netherlands

**Keywords:** Goal setting, Patient-centred care, Geriatric rehabilitation, Aged people, Functional assessment

## Abstract

**Background:**

Patient-centred goal setting is regarded as a beneficial intervention for geriatric rehabilitation. Nevertheless, its known laborious implementation in clinical practice remains an ongoing challenge. To improve implementation of patient-centred goal setting, the integration of goal setting with standardized measures has been proposed. Our objective of the current study was to explore the feasibility of Collaborative Functional Goal Setting (CFGS), i.e., using standardized functional measures to set and evaluate functional goals during geriatric rehabilitation.

**Materials and methods:**

Three medical professionals working in two geriatric rehabilitation wards were trained in CFGS and interviewed at the end of the study. We aimed at including 20 patients who underwent the CFGS intervention and could participate in open interviews. Both interviews of the professionals and patients were qualitatively analyzed.

**Results:**

Eight patients were included in the study, five of which could be interviewed. Both patients and professionals expressed a need for patient-centred goal setting. Patients indicated that goals were mainly set by the professional and that a rehabilitation plan was either not presented or its content was not clear to them. In contrast, the professionals regarded CFGS as patient-centred and potentially helpful in facilitating the goal-setting process. Nevertheless, the professionals indicated having difficulty with the implementation of the intervention.

**Conclusion:**

In the current study, we demonstrated that patient-centred goal setting supported by functional measurements was not feasible in its present form which confirms the evidence from the literature that is difficult to perform patient-centred goal setting in clinical practice.

## Introduction

Patient-centred goal setting is an important element of geriatric rehabilitation. First, it is important for clinicians to involve patients in goal setting and to respect the values and preferences of patients, in other words the patient autonomy [1, 2]. Second, goal setting might improve allocation of scarce resources to gain optimal recovery for all rehabilitation patients [1, 3]. Finally, according to a recent Cochrane review, structured goal setting in adult rehabilitation can result in higher levels of motivation, self-efficacy and health-related quality of life [4]. Although the beneficial effects of patient-centred goal setting are widely recognized, its implementation in clinical practice remains an ongoing challenge [5, 6]. According to the professionals, this may be even more difficult when it involves geriatric patients, as they often find it difficult to shape and discuss their personal rehabilitation programme and need guidance in defining their rehabilitation goals [7, 8].

Several studies have suggested the use of a measurement instrument to facilitate the goal setting process and its implementation in the clinical field [9–11]. Following this suggestion, we developed a new structured goal setting intervention called Collaborative Functional Goal Setting (CFGS), in which the patient and the professional jointly set rehabilitation goals that can be assessed and evaluated by a standardized functional measurement instrument. We hypothesized that this approach would facilitate the process of goal setting and might, therefore, improve its feasibility. Before setting up a clinical trial to investigate the validity and effectiveness of CFGS it was important to first test the feasibility of the intervention in a geriatric rehabilitation population. We subsequently conducted a qualitative pilot study to explore the feasibility of CFGS.

The objective of this study was to explore the feasibility of *Collaborative Functional Goal Setting* by exploring the views and experiences of both patients and professionals with the intervention during inpatient geriatric rehabilitation.

## Materials and methods

### The intervention

The CFGS method consisted of a step-by-step approach to structure the goal-setting process to integrate the patient’s personal rehabilitation goals into measurable standardized functional goals (see Table 1). Two functional instruments were used for this purpose: the Barthel Index and the Utrecht Scale for Evaluation of Rehabilitation [12, 13]. Prior to the study, two elderly care physicians and one nurse practitioner, working exclusively in geriatric rehabilitation, were trained. The training was provided by two experts: an educational psychologist and a geriatric rehabilitation physician, in two 4-h sessions. In this training the design of the study as well as the procedure of the intervention were explained. In addition, the participating professionals extensively practiced the intervention in role playing games and in their own clinic before the actual start of the study. During the two training sessions, personal instruction and feedback were given to the professionals.

**Table 1:** Description of the Collaborative Functional Goal Setting (CFGS) intervention

1. Within 48 h of admission a Barthel Index (BI) or the functional items of the Utrecht Scale for Evaluation of Rehabilitation (fUSER) are completed by the designated nurse
2. During the first multidisciplinary meeting (MDM), the test scores from the instrument are presented to all members of the multidisciplinary team
3. The multidisciplinary team set attainable functional goals. These goals will be presented as target scores on corresponding items of the BI or fUSER by the physician or the nurse practitioner
4. The MDM is followed by a goal-setting meeting with the patient and the physician or the nurse practitioner. Here, the patient is invited to set their own personal functional goals
5. Shared decision-making in defining the patient’s goals is ensured by the following steps
(a) Elicit the patient’s views on the degree of involvement in decision-making
(b) Patient and doctor jointly set functional goals based on consensus
(c) The physician translates these goals into target scores on the corresponding items of the functional instruments (BI or fUSER)
6. Prior to every 2 weekly MDM, a new functional assessment is conducted by the designated and presented.
7. During these meetings, the functional goals and assessment target scores will be reviewed. There are three possible actions per target score
(a) No adjustment of the target score is required or possible
(b) A higher target score is proposed to the patient
(c) A lower target score is proposed to the patient
8. The physician or the nurse practitioner informs the patient about the outcome of the MDM, specifically on the status of the functional rehabilitation goals. Furthermore, the proposed alterations in functional goals are discussed and agreed upon
9. When all potentially adjusted goals are met, the patient can potentially be discharged from the rehabilitation programme if there are no other complicating factors

### Setting and study subjects

Two geriatric rehabilitation wards, with a capacity of 40 patients, participated in this feasibility study. The three professionals were responsible for the implementation of the intervention on their ward. In one ward, CFGS was either conducted by the nurse practitioner or the physician. The other physician exclusively conducted CFGS in the other ward. We specifically focused on geriatric stroke rehabilitation patients because we wished to test this new intervention in challenging conditions, such as in patients with a high incidence of cognitive and communicative problems. We expected that we had to include a maximum of 20 patients to achieve data saturation in our qualitative analysis. To achieve an inclusion of 20 patients in a period of 6 months, we had to include 25% of the newly admitted eligible patients.

All newly admitted rehabilitation inpatients with a stroke diagnosis were asked to participate. Exclusion criteria were: inability to sign informed consent, decisional incapacity as judged by the attending physician, or mastery of the Dutch language. The primary researcher provided the patients with written and oral information about the study. Patients were included in the study once a signed informed consent was obtained. The study was approved by the Medical Ethics Review Board of the VUmc University Medical Center, Amsterdam, The Netherlands (no. FWA00017598).

### Study design

Open in-depth interviews with both the patients and professionals working with this new intervention were conducted and qualitatively analyzed. All interviews started with a grand tour question: “What can you tell me about the intervention in which you participated?” (Physicians) or “What did you notice of the intervention where physicians were trained in making patient-centred rehabilitation goals?” (Patients). This was followed by asking neutral open-ended questions and elaborating on the answers. Finally, a topic list (Table 2) was used during the interviews, which was constructed on the basis of theoretical aspects of feasibility [14]. The patients were interviewed after completion of the intramural rehabilitation programme and the professionals were interviewed at the end of the study by the first author. A summary of the findings for patients and professionals are presented separately in the Results section below.

**Table 2:** Feasibility topics and corresponding definitions. Adopted from Bowen et al. [14]

Topic	Definitions	Interviews
Acceptability	The way in which the participants respond to the intervention and the extent to which the intervention is considered suitable, satisfying or attractive	Patients and professional
Demand	The need for the intervention, along with its estimated use and perceived positive and negative effects	Patients and professional
Implementation	The extent to which the intervention can be fully implemented as proposed in an uncontrolled setting	Professional
Practicality	The extent to which the intervention can be delivered when resources, time and commitment are limited in some way	Patients and professional
Adaptation	The change required to make the intervention content more appropriate to a new situation	Professional

### Data analysis

All interview transcripts were independently analyzed by two researchers (ES, HB). First, two researchers (ES, HB) independently wrote a memo, summarizing the most important findings of the each interview. Second, after completion, all memos were checked, compared and discussed by the two researchers. In case of any discrepancies between the memos, a consensus was reached by discussion. The memo served as a tool to enhance the common understanding of the interviews by both researchers. Third, the two researchers (ES, HB) independently highlighted the sections which revealed information about feasibility of the intervention. Fourth, these sections were compared and discussed by the two researchers and consensus was reached about which segments actually revealed information about the feasibility. Fifth, every selected text segment was independently assigned to the appropriated feasibility topic. Finally, both researchers illustrated the key findings with the most representative quotations per feasibility topic which in turn were discussed with co-authors to check whether the selected quotations provided the information to support the key findings. The findings per feasibility topic are presented separately for patients and professionals in the result section. The key findings are illustrated with quotations from interviews with both patient and professionals. Quotations were translated from Dutch to English and minor editing was conducted to correct grammatical errors and improve clarity.

## Results

### Participant characteristics

From 1 January 2015 to 1 August 2015, two elderly care physicians and one nurse practitioner participated in the study and CFGS was tested on eight geriatric stroke rehabilitation patients.

Interviews were held with all three professionals and five out of the eight patients. Demographic and medical characteristics of all included patients are shown in Table 3. Drop out from the interview occurred for various reasons: one patient withdrew because of a disabling aphasia, one patient had a recurrent stroke during the study period and was subsequently admitted to the intensive care unit, and one patient could not be contacted after discharge.

**Table 3:** Patient characteristics

Name	Gender	Age	Stroke type/location	Comorbidity	Length of stay (days)	MMSE (0–30)	Interview
P1	Male	87	Ischemic stroke, right hemisphere	Atrial fibrillation, colon cancer, hypercholesterolemia	16	30	Yes
P2	Male	78	Haemorrhagic stroke, right hemisphere	Hypertension, alcohol abuse, prior ischemic stroke left hemisphere	77	28	Yes
P3	Male	73	Haemorrhagic stroke, right hemisphere	NIDDM, coronary sclerosis, TIA	45	30	Yes
P4	Female	80	Haemorrhagic stroke, brainstem	NIDDM, hypertension, atrial fibrillation, TIA	37	30	Yes
P5	Female	82	Haemorrhagic stroke, right hemisphere	Deep venous thrombosis, pulmonary embolism	27	29	Yes
P6	Male	63	Subarachnoid bleeding	Hypertension, alcohol abuse	43	30	No
P7	Male	78	Haemorrhagic stroke, left hemisphere	Prior ischemic stroke left hemisphere, coronary sclerosis, hypertension	55	Missing (refused)	No
P8	Female	74	Haemorrhagic stroke, left hemisphere	Hypertension, NIDDM, lung cancer	27	26	No

### Patient findings

The patients indicated that it was mainly the professionals who set the rehabilitation goals and that a rehabilitation plan was either not presented to them or the content of the plan was not clear. At the same time, the patients specifically stated that they wished to be actively involved in the goal-setting process, and that rehabilitation goals ought to be discussed with them. However, the extent to which they wanted to be involved in the goal-setting process varied. The patients expressed the wish for a more active role in the goal-setting process and some suggested that it should start with their own ideas about the rehabilitation process in general. Others suggested a preference for an approach in which the professional set the goals and the patient had the option to agree or disagree with these goals.

Key patients’ citations:

“I have not seen a real rehabilitation plan. For example, in the exercise room you had to practice cycling and then the physical therapist proudly said, “we made it even harder”. […] Apparently something was evaluated only the usefulness was unclear to me” (78-year-old male, with right haemorrhagic stroke).

“My advice to the professionals would be to listen carefully to the ideas that the patient has about the rehabilitation. I understand that the patient is not a professional, still you should listen carefully to the patient in question what he wants and wishes. Well, of course, in the context of the things that are professionally possible” (73-year-old male, with right haemorrhagic stroke).

“The goals and exercises were set by the therapist. I agreed to these goals and exercises if they seemed logical…” (87-year-old male, with right ischemic stroke).

### Professional findings

The professionals emphasized the relevance of patient-centred goal setting in geriatric rehabilitation. They regarded CFGS as patient centred and potentially helpful in facilitating the goal-setting process. The use of the functional instrument was considered particularly supportive in setting and evaluating rehabilitation goals. Nevertheless, the professionals found the implementation of the intervention difficult for several reasons. First, they acknowledged that the intervention differed from their conventional way of working and signalled a tendency to fall back on old routines. Second, the professionals stated that it was difficult for them to lead the rest of the multidisciplinary team in working according to the CFGS method because they had not built up extensive experience with CFGS. Finally, they reported to have filled in the functional instrument in collaboration with the patient, rather than leaving this to the nurse as was intended (see Table 1).

The professionals had several suggestions that could enhance the feasibility of CFGS. The first suggestion was to train the entire multidisciplinary team and thereby support a more multidisciplinary approach to the intervention. Second, it was suggested that the training itself should be repeated over time. However, no changes were suggested for the content of the intervention itself.

Key professionals’ citations:

“Patient-centred goal setting was facilitated by a scale such as the Utrecht Scale for Evaluation of Rehabilitation because it made clear to the patient and family what things are improving and which goals need to improve. That makes communication about the goals more clear and effective” (male elderly care physician).

“I have to admit that, because of a lack of experience, I was not able to lead the rest of the multidisciplinary team in working according to CFGS method.” (female elderly care physician).

“Because the intervention differs from daily routine, you easily fall back into old behaviour and patterns, by all sorts of things say the harshness of the day. […] On some occasions I have filed in the instrument together with the patient. In retrospect, I understood that this was not correct.” (male elderly care physician).

## Discussion

The present study was set up to test the feasibility of a new patient-centred goal-setting method for geriatric rehabilitation. The study shows that professionals considered CFGS a potential beneficial patient-centred goal setting intervention, still they found it difficult to execute and apply it into their daily routine. As a consequence, the execution and implementation was not sufficiently noticeable for the patients themselves. In conclusion, we demonstrated that patient-centred goal setting supported by functional measurements was not feasible in its present form.

### Patient-centred goal setting

In line with previous studies, the patients in the current study expressed that they wanted to be actively involved in the goal-setting process, as well as be informed about the progress of their rehabilitation [6]. However, the extent to which they wanted to be involved differed. This supports a flexible and individual-based goal-setting procedure rather than a more one-size-fits-all approach. This is in accordance with the findings of a recent systematic review that identified barriers and facilitators to goal setting during rehabilitation, which concluded that the process of goal setting should be tailored to individual patient needs and preferences, both of which could change over time [15]. CFGS incorporates this flexible approach to goal setting explicitly in the fifth step (see Table 1, Step 5a). In conclusion, patients and professionals both expressed a need for patient-centred goal setting, with the professionals especially considering CGFS to be an acceptable intervention.

### Implementation

There were several indications that CFGS was not successfully implemented. First, the professionals indicated that there was a tendency to fall back on old routines that differed from CFGS. In particular, the professionals stated that CFGS was not always systematically and accurately executed as intended. Finally, the patients had not noticed the intended patient-centredness of CFGS. The literature describes similar difficulties with patient-centred goal setting in rehabilitation patients [6]. Importantly, the literature further shows that goal setting is generally new to patients and that they, therefore, have difficulty understanding what is expected of them [6]. This might be an alternative explanation for why patients in the current study did not perceive the intervention to be patient centred.

### Limitation

The major limitation of our study was that it was tested on a limited number of patients. This might have negatively influenced the implementation process, especially because the professionals reported that it was difficult to build more comprehensive experience with the intervention. In spite of this limitation, we reached the data saturation point to make a conclusion on feasibility of the intervention. First, the professionals stated having difficulties with the implementation and execution of the intervention. In addition, all the participating patients uniformly report the same experience and stated that the execution of CFGS was not clear to them. Because of the ongoing difficulties in executing and implementing the intervention, we reached a point where it seemed unethical to continue and include more patients into the study.

### Clinical implication

Despite this limitation, we conclude that both professionals and patients experience the need for patient-centred goal-setting interventions in geriatric rehabilitation. However, in the current study, both patients and professionals raised considerable concerns about the feasibility of CFGS. The professionals made a number of recommendations for improving its feasibility. First, the entire multidisciplinary team needs to be trained in CFGS to ensure a uniform and multidisciplinary approach. Second, CFGS training should be regularly updated so it becomes a familiar daily routine. Third, because CFGS differs from current daily routines, and experience is needed to master the skills to implement the intervention, sufficient time and resources must be made available for its implementation.
